# 

**Published:** 1915-07-24

**Authors:** 


### CONTENTS.

| | PAGE |
|---|---|
| **The Organisation of Military Medical Services** | 341 |
| **Women Students at Charing Cross Hospital** | 342 |
| **Hospital and Institutional News ... ... ...** | 343 |
| Hospitals and the Spirit Duties : The Effect of the Act—The British Hospitals Association : Conversation on July 30—The Wages of Ambulance Drivers—The War's Effect on the Health of School Children—Relation of School Doctor to Teacher : A Regrettable Incident—A Lesson for Cambridge School Managers—Sir Frederick Treves—The Empire Hospital : For Officers—Secretary of Stockport Infirmary Resigns—Major Tyler and the Vacancy at Stockport—Saving on the Tuberculosis Service—Professor Denys of Louvain on Tuberculosis—Poor-Law Infirmaries and Coal Contracts—Dispensaries and the Incidence of Tuberculosis—"Places of Safety" under the Mental Deficiency Act—Insurance Practitioners and the War—The "Bart.'s" Hospital Badge—Serious Charge Against a Doctor—Why the Charge was Dismissed—The War and the Site of the New Bradford Royal Infirmary—An Army Pharmaceutical Service—This Week's Drug Market. | |
| **Medical Service in the Highlands and Islands** | 349 |
| Life and Needs of the Remote Doctors. | |
| **Notes by a Surgeon on Active Service... ...** | 351 |
| Circumcision Under Local Anæsthesia—Simple Treatment of Scabies—Venereal Disease—Dust and Smoke Conjunctivitis—The Importance of Good Boots. | |
| **The Cause of Poor-Law Reform ... ... ...** | 352 |
| Two Typical Needs and the Remedy : The Local Government Inquiry at Epping—The Local Government Board's Decision and the Guardians' Action—Guardians and Able-Bodied Paupers. | |
| **Medical Appointments ... ... ... ...** | 352 |

[*See over.*]

### CONTENTS—(*continued*).

| | PAGE |
|---|---|
| **A Medical Causerie** ... ... ... ... ... | 353 |
| **Round the World** ... ... ... ... ... | 354 |
| Fellow-Workers and the Roll of Honour. | |
| **National Insurance Act Developments** ... ... | 355 |
| The Parliamentary Debate on the Cost of National Insurance. | |
| **The Hanbury Medallist** .. ... ... ... | 356 |
| **An Indian Hospital Ship** (*illustrated*) ... ... | 357 |
| Voyages and Equipment of the Hospital Ship *Madras*. | |
| **The Editor's Letter-Box** ... ... ... ... | 359 |
| Hospitals and the Spirit Tax. | |
| Hospitals and the Spirit Duties. | |
| Pickled Eggs—and Preserving Jam. | |
| The Hospital Egg : Merchants' Views. | |
| **The Book Market** ... ... ... ... ... | 360 |
| **Legal Notes for Institutional Workers** ... | 361 |
| A Suggested Public Health Improvement. | |
| The Fees of Medical "Expert" Witnesses. | |
| **Dispensing for Women** ... ... ... ... | 361 |
| **A Red Cross Memorial** ... ... ... ... | 362 |
| **Institutional Needs** ... ... ... ... ... | 362 |
| The "Buffalo" Meat and Vegetable Chopper. | |
| A New Bandage-Winder. | |
| **Latest Installations in Hospital Equipment** ... | 362 |
| What Others Are Doing. | |
| **Personalia** ... ... ... ... ... ... | 362 |
| **The Institutional Worker** ... ... (Suppl.) | 1 |
| The Admission of In-Patients : Answer to June Question. | |
| On Engaging Clerks. | |
| Question for July. | |
| Enquiries and Answers. | |
| The Sick and In Need : Home for Epileptic Woman—Home for Chronic Cripple Boy. | |
| Employment and Training—Nursing in America or Canada—School Nursing—Training at Eighteen—Training at Nineteen in Ireland—Free Maternity Training—Children's Lady Nurse. | |

